# miR-128-3p Regulates Follicular Granulosa Cell Proliferation and Apoptosis by Targeting the Growth Hormone Secretagogue Receptor

**DOI:** 10.3390/ijms25052720

**Published:** 2024-02-27

**Authors:** Shucan Dong, Shengwei Jiang, Biwei Hou, Yaokun Li, Baoli Sun, Yongqing Guo, Ming Deng, Dewu Liu, Guangbin Liu

**Affiliations:** Guangdong Laboratory for Lingnan Modern Agriculture, College of Animal Science, South China Agricultural University, Guangzhou 510642, China; scdong@stu.scau.edu.cn (S.D.); jsw1678693227@126.com (S.J.); 20213139038@stu.scau.edu.cn (B.H.); ykli@scau.edu.cn (Y.L.); baolisun@scau.edu.cn (B.S.); yongqing@scau.edu.cn (Y.G.); dengming@scau.edu.cn (M.D.)

**Keywords:** miR-128-3p, granulosa cells, proliferation, apoptosis, follicular development

## Abstract

The proliferation and apoptosis of granulosa cells (GCs) affect follicle development and reproductive disorders, with microRNAs playing a crucial regulatory role. Previous studies have shown the differential expression of miR-128-3p at different stages of goat follicle development, which suggests its potential regulatory role in follicle development. In this study, through the Cell Counting Kit-8 assay, the EDU assay, flow cytometry, quantitative real-time polymerase chain reaction, Western blot, and the dual-luciferase reporter assay, we used immortal human ovarian granulosa tumor cell line (KGN) cells as materials to investigate the effects of miR-128-3p and its predicted target gene growth hormone secretagogue receptor (GHSR) on GC proliferation and apoptosis. The results show that overexpression of miR-128-3p inhibited the proliferation of KGN cells, promoted cell apoptosis, and suppressed the expression of proliferating cell nuclear antigen (PCNA) and B-cell lymphoma-2 (BCL2) while promoting that of Bcl-2 associated X protein (BAX). The dual-luciferase reporter assay revealed that miR-128-3p bound to the 3′ untranslated region sequence of GHSR, which resulted in the inhibited expression of GHSR protein. Investigation of the effects of GHSR on GC proliferation and apoptosis revealed that GHSR overexpression promoted the expression of PCNA and BCL2, enhanced GC proliferation, and inhibited cell apoptosis, whereas the opposite effects were observed when GHSR expression was inhibited. In addition, miR-128-3p and GHSR can influence the expression of extracellular signal-regulated kinase 1/2 protein. In conclusion, miR-128-3p inhibits KGN cell proliferation and promotes cell apoptosis by downregulating the expression of the GHSR gene.

## 1. Introduction

Follicular development is related to animal reproduction and has piqued considerable research interest. Ovarian follicles comprise theca cells (TCs), granulosa cells (GCs), and oocytes and are widely considered a functional unit of the ovary for reproductive function [1]. The proliferation, apoptosis, and differentiation of GCs affect the fate of follicles [2,3]. GCs occupy the middle layer between TCs and oocytes, and they can influence the quality of oocytes by signaling and exchanging substances with them through gap junctions and cooperating with TCs to produce steroid hormones [4,5]. GCs can also influence the growth of surrounding follicles through autocrine and paracrine secretion [6]. Mammals possess large pools of primitive follicle reserves; however, the ovulation efficiency of follicles is inefficient; less than 1% of follicles can ovulate during follicle development, and the rest of the follicles remain in atresia [7]. Apoptosis of GCs is an important cause of follicular atresia [2,8,9]. Apoptosis of GCs is regulated by various factors, including gene expression, endocrine hormones, and epigenetic modifications [10,11,12].

Past studies have extensively explored gene expression during follicle development and revealed important signaling pathways that regulate follicle development [13]. Noncoding RNAs, as important regulators of gene expression, show a close relation to the life activities of plants and animals [14]. However, exploration of the regulation of gene expression by these factors remains incomplete. MicroRNAs (miRNAs) have been widely recognized as regulatory factors of gene expression because they can modulate the stability and translation of messenger RNA (mRNA) and thereby influence gene protein expressions [15,16]. MiRNAs are essential for the reproduction of female animals [17]. They can regulate the synthesis of ovarian steroid hormones and participate in the proliferation, differentiation, and apoptosis of follicular cells [18,19,20]. miR-128-3p refers to a conserved RNA sequence that can regulate the proliferation, apoptosis, and disease occurrence among various tissues and cells [21,22,23]. miR-128-3p is expressed in ovarian follicles, with low and high expressions in small and large follicles, respectively [24]. Furthermore, compared with that in young women with normal ovarian reserves, the expression of miR-128-3p is considerably upregulated in young women with diminished ovarian reserves [25]. These findings suggest the possible important role of miR-128-3p in the process of follicular development.

Follicle development refers to a dynamic process involving the continuously changing expressions of many genes to regulate follicle growth. Previous transcriptomic studies have revealed that compared with that in small follicles, the mRNA expression level of the *growth hormone secretagogue receptor* (*GHSR*) gene is substantially decreased in large follicles [24]. This condition suggests that *GHSR* may play a regulatory role in the process of follicle development. The *GHSR* gene is widely expressed in various animal tissues and participates in the regulation of food intake, energy metabolism, cardiovascular function, cell proliferation, and reproduction [26,27,28,29,30]. Ghrelin is the endogenous ligand for GHSR and plays a role in the reproductive processes of animals [31]. The administration of ghrelin in vivo can cause changes in the morphology and structure of rat ovaries, which result in ovarian atrophy and induce cell apoptosis [32]. Ghrelin also increases phosphor extracellular signal-regulated kinase (ERK) 1/2 levels and cell proliferation in cultured GCs in a GHSR1a-dependent manner and increases the expressions of estradiol and aromatase activity in GCs [31,33]. These studies demonstrated the crucial role of GHSR in follicle development. Investigation of the regulation of GHSR expression is crucial for a comprehensive understanding of follicular development. However, there is currently no research reporting which genes regulate the expression of the GHSR gene during follicle development. miRNAs can regulate gene expression by targeting the 3′ untranslated region (3′UTR) of mRNA. So, it is speculated that the expression of the *GHSR* gene is regulated by miRNA. Exploration of the miRNAs that regulate the *GHSR* gene will contribute to revealing its regulatory network and its role in follicular development.

Investigating the genes that influence granulosa cell proliferation and apoptosis is of significant importance in regulating follicular development and addressing follicular diseases. Therefore, this study focuses on exploring the relationship between miR-128-3p and the *GHSR* gene, as well as their expression’s impact on granulosa cell proliferation and apoptosis, aiming to provide potential molecular targets for regulating follicular development.

## 2. Results

### 2.1. Prediction of miR-128-3p Target Genes and Functional Enrichment Analysis of Its Target Genes

**Figure 1:**
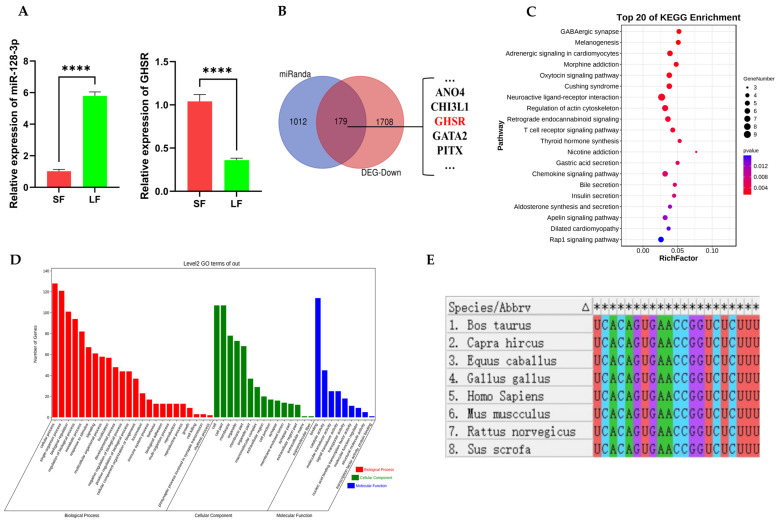
Functional enrichment analysis of predicted target genes for miR-128-3p. (**A**) Relative expression levels of miR-128-3p and GHSR genes in small and large follicles validated via qRT-PCR. (**B**) Venn diagram analysis of predicted target genes and downregulated differentially expressed genes in large follicles. (**C**) Enrichment analysis of KEGG pathways for predicted target genes. (**D**) Enrichment analysis of GO functions for predicted target genes. (**E**) Conservation analysis of miR-128-3p sequences in eight species, “*” represents the consistent ribonucleic acid (RNA) base sequence in the column. Data are presented as mean ± standard deviation (SD); **** *p* < 0.0001.

RNA-sequencing of large and small follicles of Leizhou goat showed the differential expression of miR-128-3p in large and small follicles, with a significantly higher differential expression in large follicles than in small follicles [24]. Quantitative real-time polymerase chain reaction (qRT-PCR) results indicate that the expression patterns of miR-128-3p and *GHSR* gene in goat ovarian follicles are consistent with the sequencing findings reported by Feng et al. (Figure 1A). Using the Miranda database, we predicted 1191 target genes of miR-128-3p. The classic mechanism of miRNA suggests that miRNAs inhibit the stability or translation efficiency of target gene mRNAs by binding to their 3′UTR through seed sequence complementarity. On this basis, we performed Venn analysis on predicted target genes and considerably downregulated mRNAs in large follicles of Leizhou goats based on sequencing data. A total of 179 common genes were identified (Figure 1B). Functional enrichment analysis of these 179 genes was performed using the Kyoto Encyclopedia of Genes and Genomes (KEGG) and Gene Ontology (GO) to explore the possible functions of miR-128-3p in follicular development. KEGG results show the significant enrichment of these target genes in the endocrine system, signal transduction pathways, the nervous system, and the digestive system. These genes particularly exhibit enrichment in the endocrine system and signal transduction pathways, including melanogenesis, insulin secretion, thyroid hormone synthesis, ovarian steroidogenesis, the cGMP-PKG signaling pathway, the WNT signaling pathway, the cAMP signaling pathway, etc. (Figure 1C). GO enrichment analysis revealed the most significant biological process, cellular component, and molecular function terms, namely, embryonic placenta development, the cell junction, and chloride channel activity, respectively. In addition, these target genes are significantly enriched in cell proliferation, cell migration, mitogen-activated protein kinase cascade, and ERK1 and ERK2 cascades (Figure 1D). Therefore, miR-128-3p possibly plays an important role in animal follicle development and may be involved in the regulation of cell proliferation and apoptosis.

MEGA 7.0 software was used to conduct sequence similarity analysis of miR-128-3p sequences in eight common mammalian species, including humans, goats, pigs, horses, cows, chickens, mice, and rats. The results reveal that the miR-128-3p sequences are completely identical across these mammalian species (Figure 1E), which suggests a high level of conservation and possible conserved functions of miR-128-3p in mammals.

### 2.2. MiR-128-3p Is Involved in GC Apoptosis and Proliferation

**Figure 2:**
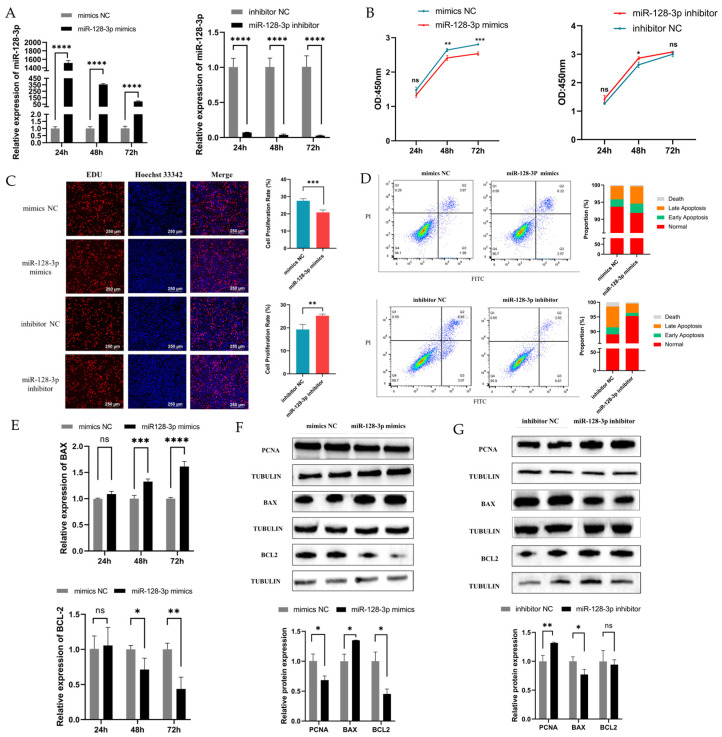
Regulation of GC proliferation and apoptosis by miR-128-3p. (**A**) Relative expression levels of miR-128-3p after transfection of miR-128-3p mimics and inhibitors into GCs detected by qRT-PCR. (**B**,**C**) Effect of miR-128-3p mimics and inhibitor on GC proliferation shown by CCK-8 and EDU assay. (**D**) Apoptosis rate of GCs measured via flow cytometry. (**E**) Relative mRNA expression levels of *BAX* and *BCL2* genes. (**F**,**G**) Effects of miR-128-3P on PCNA, BAX, and BCL2 protein expressions in GCs via Western blot, respectively. Data are presented as mean ± SD; ns, no significance; * *p* < 0.05; ** *p* < 0.01; *** *p* < 0.001; **** *p* < 0.0001.

To investigate the effect of miR-128-3p on the proliferation and apoptosis of GCs, we conducted transfection experiments using exogenous miR-128-3p mimics and inhibitors in immortal human ovarian granulosa tumor cell line (KGN) cells. The expression levels of miR-128-3p increased and decreased upon transfection with mimics and inhibitors, respectively (Figure 2A). In addition, Cell Counting Kit-8 (CCK8) analysis revealed the considerably reduced viability of KGN cells at 48 and 72 h post-transfection with miR-128-3p mimics. Conversely, the proliferation viability of cells substantially increased at 48 h following transfection with miR-128-3p inhibitors. However, no significant difference was observed at 24 and 72 h (Figure 2B). Similar to the CCK-8 results, the EDU assay indicated that the proliferation of KGN cells was inhibited by miR-128-3p at 48 h (Figure 2C). The effect of miR-128-3p on apoptosis was examined via flow cytometry. Flow cytometry results show that compared with that in the control group, the apoptosis rate was increased in the miR-128-3p overexpression group and decreased in the suppression group (Figure 2D). In addition, miR-128-3p mimics remarkably downregulated the expression level of the antiapoptotic *B-cell lymphoma-2* (*BCL2*) gene and upregulated the mRNA expression level of the proapoptotic *Bcl-2 associated X protein* (*BAX*) gene and *BAX*/*BCL2* ratio in KGN cells (Figure 2E). Western blot results revealed that overexpression of miR-128-3p greatly increased the protein level of BAX and suppressed the expression of proliferating cell nuclear antigen (PCNA) and BCL2 protein in KGN cells (Figure 2F). On the contrary, inhibition of miR-128-3p expression resulted in decreased protein expression of BAX and increased the protein level of PCNA in KGN cells, but that of BCL2 protein was unaffected (Figure 2G). In general, these results suggest that miR-128-3p may inhibit the proliferation of KGN cells and promote their apoptosis.

### 2.3. GHSR Is a Target Gene of miR-128-3p

**Figure 3:**
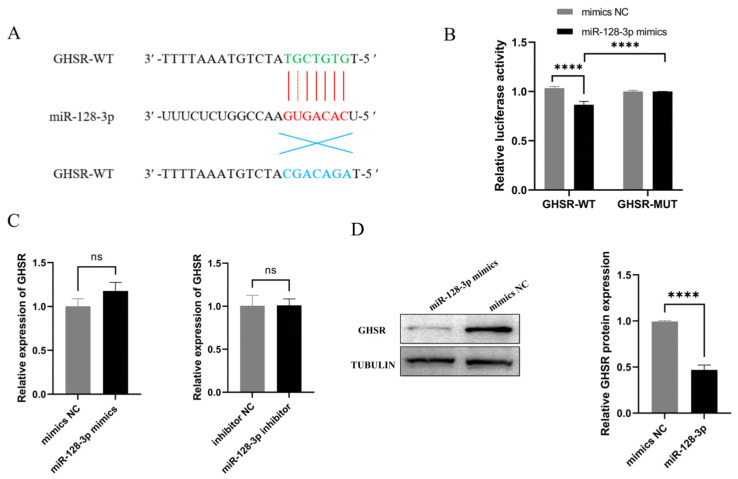
Analysis of the targeting relationship between miR-128-3p and its predicted target gene *GHSR*. (**A**) Schematic representation of the interaction between miR-128-3p and wild-type (green) and mutant (blue) 3′UTR regions of *GHSR*, with the miR-128-3p seed sequence highlighted in red. (**B**) Dual-luciferase reporter assay of the regulatory relationship between miR-128-3p and predicted binding sites on GHSR. (**C**) Effect of miR-128-3p mimics and inhibitor on *GHSR* gene mRNA levels. (**D**) Effects of miR-128-3p overexpression on GHSR protein expression. Data are presented as mean ± SD; ns, no significance; **** *p* < 0.0001.

Venn diagram analysis of target genes predicted by miRNA and differentially expressed genes in large and small follicles of Leizhou goat predicted GHSR and other genes as miR-128-3p target genes. RNAhybrid database analysis showed that the miR-128-3p seed sequence can bind to the 3′UTR region of GHSR (Figure 3A). To determine whether miR-128-3p targets the GHSR gene, we constructed luciferase reporter plasmids containing the GHSR-predicted binding site or mutation and cotransfected them with miR-128-3p mimics into 293Ts. Luciferase activity was measured 48 h after transfection. miR-128-3p significantly reduced the luciferase activity of the reporter containing the GHSR-predicted binding site fragment, and no change in the reporter activity was observed when using the GHSR-predicted binding site fragment mutant. These findings indicate that *GHSR* is a direct target of miR-128-3p (Figure 3B). Furthermore, miR-128-3p mimics significantly suppressed GHSR protein expression but did not affect changes in *GHSR* mRNA levels (Figure 3C,D). Overall, these results imply that miR-128-3p directly targets GHSR and inhibits its protein expression.

### 2.4. GHSR Is Involved in GC Apoptosis and Proliferation

**Figure 4:**
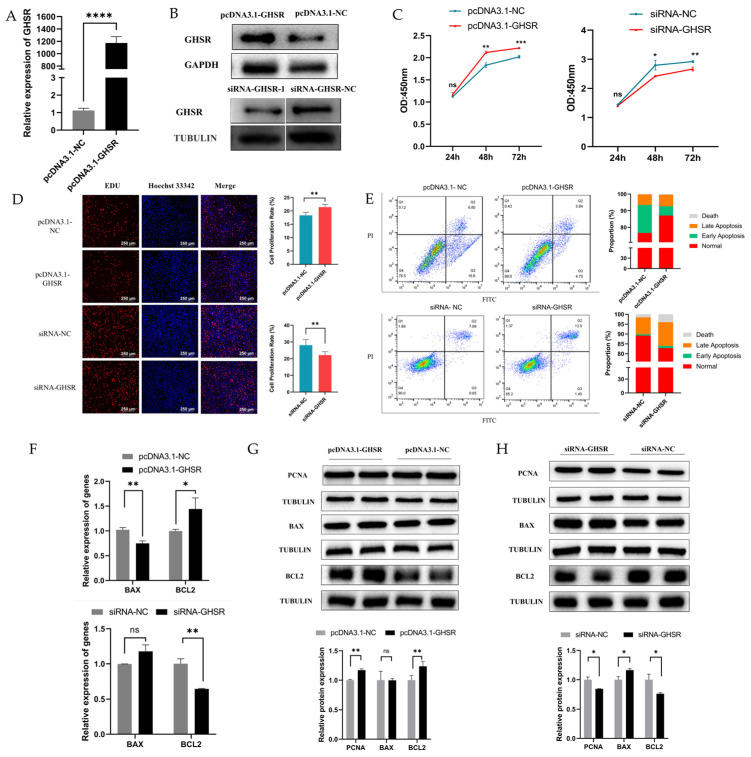
GHSR regulates GC proliferation and apoptosis. (**A**) Relative expression levels of *GHSR* after transfection into GCs using qRT-PCR. (**B**) Western blots of GHSR protein expression after overexpression and inhibition of GHSR. (**C**,**D**) GC proliferation after overexpression and inhibition of GHSR through CCK-8 and EDU assay. (**E**) Apoptosis rate of GCs measured via flow cytometry. (**F**) Relative mRNA expression levels of *BAX* and *BCL2* genes. (**G**,**H**) Effects of overexpression and inhibition of GHSR on PCNA, BAX, and BCL2 protein expression in GCs determined by Western blot. Data are presented as mean ± SD; ns, no significance; * *p* < 0.05; ** *p* < 0.01; *** *p* < 0.001; **** *p* < 0.0001.

To determine the function of GHSR in the proliferation and apoptosis of KGN cells, we overexpressed and knocked down GHSR in KGN cells using an overexpression vector and small interfering RNA (siRNA) and observed the remarkably increased and decreased transcript and protein levels of GHSR, respectively (Figure 4A,B). CCK8 and EDU assay showed that overexpression or knockdown of GHSR significantly promoted and inhibited cell proliferation, respectively (Figure 4C,D). Flow cytometric results show that overexpression of GHSR greatly inhibited KGN cell apoptosis, whereas the opposite was true for its inhibited expression (Figure 4E). At the mRNA level, GHSR overexpression significantly decreased *BAX* expression and increased that of *BCL2*. After inhibition of the expression of GHSR, the expression of the *BAX* gene increased, but the difference was nonsignificant. In addition, the expression level of *BCL2* showed a significant reduction (Figure 4F). Western blot results revealed that the overexpression of GHSR can increase the PCNA and BCL2 protein levels but did not affect the BAX protein expression. After inhibition of the expression of GHSR, BAX protein expression increased significantly, and the protein level of PCNA and BCL2 notably decreased (Figure 4G,H). All these results suggest that GHSR can promote GC proliferation and inhibit apoptosis.

### 2.5. Cotransfection of GHSR and miR-128-3p Affects Apoptosis in GCs

**Figure 5:**
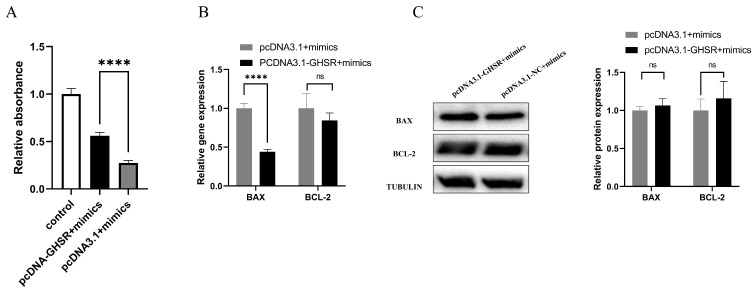
Analysis of the targeting relationship between miR-128-3p and its predicted target gene GHSR. (**A**) Compensatory expression of GHSR in KGN cells after the overexpression of miR-128-3p on cell proliferation vitality. (**B**) Influence of miR-128-3p overexpression and subsequent GHSR rescue on mRNA levels of *BAX* and *BCL2* genes in GCs. (**C**) Effects of miR-128-3p overexpression and subsequent GHSR rescue on the protein levels of BAX and BCL2 in GCs. Data are presented as mean ± SD; ns, no significance; **** *p* < 0.0001.

To investigate the effect of the interaction between miR-128-3p and GHSR on the proliferation and apoptosis of KGN cells, we conducted transfection experiments using pcDNA3.1-GHSR or pcDNA3.1 plasmids in combination with miR-128-3p mimics. Our findings demonstrate significantly higher cell proliferation in the cotransfected group with pcDNA3.1-GHSR and miR-128-3p than in the cotransfected group with pcDNA3.1 and miR-128-3p (Figure 5A). Notably, the overexpression of GHSR along with miR-128-3p transfection significantly reduced the mRNA expression level of *BAX* and the ratio of *BAX*/*BCL2*, but no difference was detected in the protein expression levels between BAX and BCL2 (Figure 5B,C). These results suggest that the GHSR gene can attenuate the effects of miR-128-3p by promoting apoptosis and inhibiting the proliferation of GCs.

### 2.6. miR-128-3p and GHSR Regulate ERK1/2 Protein Expression

**Figure 6:**
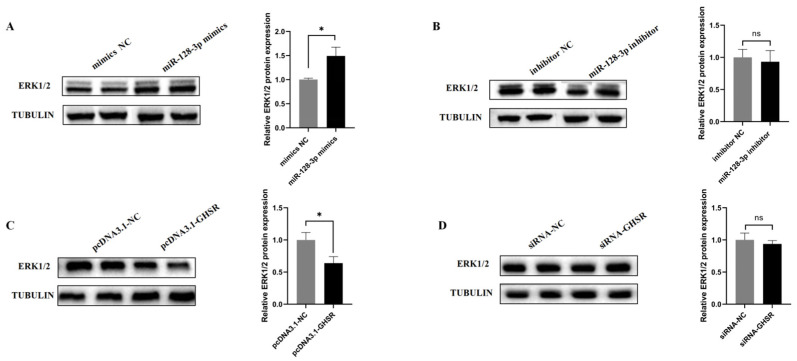
Effects of miR-128-3p and GHSR on ERK1/2 protein expression. (**A**) Influence of miR-128-3p mimics overexpression on ERK1/2 protein expression in GCs. (**B**) Influence of miR-128-3p inhibitor on ERK1/2 protein expression in GCs. (**C**) Effects of GHSR overexpression on ERK1/2 protein expression in GCs. (**D**) Influence of inhibiting GHSR expression on ERK1/2 protein expression in GCs. Data are presented as mean ± SD; ns, no significance; * *p* < 0.05.

ERK1/2 refers to important intracellular signaling molecules that regulate various cellular physiological and pathological processes and play a crucial role in the regulation of cell proliferation and apoptosis. To verify the effects of miR-128-3p and GHSR on the ERK pathway, we first used Western blot analysis to examine the effects of miR-128-3p overexpression or inhibition on the protein expressions of ERK1/2 in KGN cells. The results show that compared with the mimics NC group, the overexpression of miR-128-3p significantly upregulated the protein expression levels of ERK1/2, whereas the miR-128-3p inhibitor showed no effect (Figure 6A and Figure 6B, respectively). We then examined the effect of GHSR overexpression on the protein expression of ERK in KGN cells and observed that the protein expression levels of ERK1/2 decreased substantially, and the inhibition of GHSR expression had no effect on ERK1/2 protein expression (Figure 6A,B). These data suggest the possible involvement of miR-128-3p and GHSR in the regulation of the ERK pathway.

## 3. Discussion

Follicular development refers to a complex process in which genes in follicles constantly cause expression changes in response to follicular development. As regulators of gene expression, miRNAs play an important role in the development process. In this study, miR-128-3p inhibited the proliferation of GCs and promoted apoptosis, and the regulatory effect of GHSR on the proliferation and apoptosis of GCs was opposite that of miR-128-3p. We also observed that miR-128-3p can target the 3′UTR region of GHSR to inhibit its expression. In addition, miR-128-3p and GHSR regulated the ERK1/2 activity involved in follicular developmental regulation.

Ovaries can produce hormones that maintain the reproductive capacity of female animals. KEGG functional enrichment analysis of the predicted target gene of miR-128-3p revealed that the predicted target genes *cytochrome p450 family 11 subfamily a member 1* (*CYP11A1*), *adenylate cyclase 1* (*ADCY1*), and *adenylate cyclase 2* (*ADCY2*) of miR-128-3p can be enriched into the signaling pathway of ovarian steroid production. Production of steroid hormones, which mainly includes androgen, estrogen, progesterone, and so on, within the ovaries is essential for successful ovulation [34]. These hormones are synthesized in follicles with cholesterol as the original material [35]. The protein encoded by *CYP11A1* mainly converts cholesterol to pregnenolone, which is the first rate-limiting enzyme in steroid hormone synthesis and whose expression directly affects steroid hormone synthesis [36]. *ADCY1* and *ADCY2* belong to the adenylate cyclase family, and they mainly catalyze the conversion of adenosine-5′-triphosphate into 3′,5′-adenosine monophosphate (cyclic AMP/cAMP) [37]. cAMP is a key factor in the synergistic activity of follicle-stimulating hormone and luteinizing hormone (LH) in promoting estrogen secretion, follicle development, and ovulation [38,39]. These studies suggest the possible involvement of miR-128-3p in the secretion of steroid hormones during follicular development and its great research potential.

Follicular atresia is an important physiological process during ovarian follicle development, maturation, and ovulation [40,41]. This process is affected by a combination of factors, among which the apoptosis of granule cells serves as one of the triggers of antral follicle atresia [42,43]. miRNAs play important roles in the proliferation and apoptosis of follicular GCs [44,45]. MiR-128-3p regulates cell proliferation and apoptosis in various tissues. In this study, miR-128-3p can inhibit the proliferation of KGN cells and promote apoptosis, which is consistent with the results reported by Ning Z. et al., who observed that miR-128-3p inhibited the proliferation and promoted the apoptosis of chicken primary GCs [46]. Furthermore, the inhibition of miR-128-3p in rats can alleviate the adrenaline-induced apoptosis of GCs [47]. These results imply that the regulatory role of miR-128-3p in the proliferation and apoptosis of follicular GCs may be evolutionarily conserved. Therefore, in the late stage of follicular development, miR-128-3p may promote ovulation by inhibiting granulosa cell proliferation and promoting granulosa cell apoptosis. Additionally, upregulation of miR-128-3p has been observed in young women with premature ovarian failure, possibly due to the excessive expression of miR-128-3p inhibiting granulosa cell proliferation and leading to excessive apoptosis, causing follicular atresia and premature ovarian failure. This hypothesis requires specific experimental validation.

miRNAs can regulate the expression of target genes by influencing the stability of target gene mRNAs or blocking the translation process to exert their functions [48,49,50]. This study demonstrated that miR-128-3p in KGN cells can target GHSR to inhibit the expression of GHSR proteins. Furthermore, miR-128-3p and GHSR exhibited opposite effects on KGN cells, with GHSR promoting cell proliferation and inhibiting apoptosis. In addition, the overexpression of GHSR can alleviate the inhibitory effect of miR-128-3p on the proliferation of KGN cells. Previous transcriptomic sequencing of goat follicles revealed that compared with that in small follicles, the expression level of miR-128-3p was upregulated in large follicles, and that of the GHSR gene was downregulated [24]. This finding suggests that during follicle development, as the expression level of miR-128-3p increases, the expression of the GHSR gene is gradually suppressed, leading to the inhibition of granulosa cell proliferation. The protein encoded by GHSR, that is, GHSR1a, serves as the endogenous receptor for ghrelin. Currently, the functional role of GHSR in GCs has not been reported, with most studies focusing on the function of its endogenous ligand, which is ghrelin. A study revealed that ghrelin promoted the proliferation of porcine follicular granule cells and inhibited their apoptosis by affecting the expressions of *PCNA* and *BAX* genes [51,52]. Studies on chickens also showed that ghrelin promoted the proliferation of chicken follicle granule cells and inhibited their apoptosis through GHSR1a [53]. The observed results in KGN cells overexpressing the GHSR gene are consistent with these research findings. Therefore, it is possible to regulate the action of ghrelin hormone by modulating the expression of *GHSR* through miR-128-3p. However, further experimental validation is required to determine whether GHSR can exert its effects independently of ghrelin.

ERK1/2 plays a crucial role in mammalian follicle development. It regulates the proliferation and apoptosis of GCs and is an important component of the LH peak-induced cascade response [54,55]. Studies have shown that activation of ERK1/2 promotes cell proliferation. However, in this study, the overexpression of miR-128-3p promoted the expression of ERK1/2 proteins in KGN cells, and overexpression of GHSR inhibited their expression. This finding is in contrast to the result showing that elevated levels of ERK1/2 protein promoted cell proliferation. Moreover, in benign prostatic hyperplasia, the deficiency in neural epidermal growth factor-like like protein 2 can inhibit cell proliferation by suppressing the activation of ERK1/2 through phosphorylation [56]. In KGN cells, miR-128-3p and its target gene, GHSR, regulate the protein expression of ERK1/2. However, whether changes in ERK1/2 protein levels affect cell proliferation in this context requires further investigation.

Follicle development is regulated by a complex and intricate network, and understanding follicle development at the cellular level is hindered by various limitations. In order to gain a better understanding of the role of miR-128-3p in follicular development and its molecular mechanisms, scientists must transfect miR-128-3p into animal ovaries or construct an ovarian tissue-specific miR-128-3p knockout animal model. Subsequently, the direct impact of miR-128-3p on follicular development will be observed through tissue sectioning, immunohistochemistry, and hormone measurements. Various omics techniques such as transcriptomic sequencing, ribosome profiling, proteomics, and metabolomics will then be employed to elucidate the molecular mechanisms by which miRNA-128-3p regulates follicular development. Animal experiments will provide a visual assessment of the regulatory role of miR-128-3p in follicular development, thereby laying a theoretical foundation for a comprehensive understanding of follicular development and addressing ovarian diseases.

## 4. Materials and Methods

### 4.1. Cell Line Selection and Culture

KGN cells can respond to gonadotropins and exhibit high aromatase activity. Fas-mediated apoptosis has been observed in KGN cells. Therefore, KGN cells are considered a valuable model for the study of steroidogenesis, cell growth, and apoptosis regulation in human GCs [57,58]. Furthermore, it has been discovered that KGN cells express the GHSR gene and are responsive to stimulation by ghrelin analog growth hormone-releasing peptide-2 [59]. Therefore, this study selected KGN cells to explore the relationship between miR-128-3p and GHSR gene expression and their effects on the proliferation and apoptosis of KGN cells. Additionally, 293T cells are commonly used as a tool cell line for the investigation of dual-luciferase reporter activity. Hence, in this study, KGN and 293T cells were employed to investigate the relationship and function between miR-128-3p and the predicted target gene GHSR. They were cultured in Dulbecco’s Modified Eagle’s Medium/F12 (GIBCO, Shunyi district, Beijing, China) supplemented with 10% fetal bovine serum (Fisher, Grand Island, NY, USA) and 100 IU/mL penicillin/streptomycin (Gibco, Grand Island, NY, USA) at 37 °C in a humidified atmosphere of 5% CO_2_.

### 4.2. Vector Construction

The plasmid was constructed using seamless cloning technology, and the primers were designed based on the target sequence of the inserted vector and the sequence near the enzymatic site of the planned insert of the vector, which was synthesized by BGI (Beijing, China), using CE Design (https://crm.vazyme.com/cetool/simple.html (accessed on 9 January 2023)), an online primer design software from Vazyme Biotech Co., Ltd. (Nanjing, Jiangsu, China). The designed primers were used to amplify the target fragment with homologous arm sequences from cDNA and recover it using the TIANguick Midi Purification Kit (Tiangen, Changping district, Beijing, China). pcDNA3.1 and pmirGLO were then digested using two endonucleases, and the cleaved plasmid was recovered using the TIANguick Midi Purification Kit (Tiangen, Changping district, Beijing, China). The above-purified target fragment and linearized vector were ligated using the ClonExpress Ultra One-Step Cloning Kit (Vazyme, Nanjing, Jiangsu, China). The constructed vector was transfected into DH5α competent cells (Vazyme, Nanjing, Jiangsu, China) for screening and culture expansion, and finally, sequencing was performed to detect the successful construction of the vector. Table S1 provides detailed information on the primers used in this study.

### 4.3. Cell Transfection

Cell transfection was used to send foreign genes into cells for expression to explore gene function. miR-128-3p mimics, inhibitors, negative controls, and siRNA sets were designed and synthesized by Ribobio (Guangzhou, Guangdong, China). Supplementary Table S2 provides synthetic sequence information. The transfection operation was carried out when the cells grew to 60–80% and were in good condition. Lipofectamine^TM^ 3000 Transfection Reagent (Invitrogen, Carlsbad, CA, USA) was used to transfect miRNAs, siRNAs, and overexpression vectors into the cells in accordance with the manufacturer’s instructions. A total of 100 nM/mL siRNA, miR-128-3p mimics, and inhibitor and 2 µg/mL pcDNA3.1-GHSR and pcDNA3.1-NC plasmid were used in this study.

### 4.4. RNA Extraction and qRT-PCR Analysis

The total RNA in KGN cells was extracted using SteadyPure Universal RNA Extraction Kit (Agbio, Changsha, China) following the manufacturer’s instructions. miRNA was reverse transcribed into cDNA using miRNA 1st-Strand cDNA Synthesis Kit (by stem-loop) (Vazyme, Nanjing, Jiangsu, China) via the stem-loop method, and cDNA of mRNA was synthesized through reverse transcription using PrimeScript™ RT Master Mix (Perfect Real Time) (Takara, Kusatsu, Shiga, Japan). ChamQ SYBR qPCR Master Mix (Low ROX Premixed) (Vazyme, Nanjing, Jiangsu, China) and QuantStudio 5 real-time PCR system (Thermo Fisher Scientific, Marsiling, Singapore) were used in qRT-PCR. Melting curve analyses confirmed that all primers were specific for their respective transcript. U6 (U6 snRNA) and β-actin were used as internal controls, and the results were calculated using the 2^−ΔΔCt^ method. Experiments were carried out in triplicate. All the primers were synthesized by BGI (Beijing, China), and the complete list of primers used is shown in Table S3.

### 4.5. Cell Counting Kit-8

Cell proliferation assay was conducted using a CCK-8 kit (Biosharp, Shunyi district, Beijing, China). Approximately 2000 cells were added to each well of the 96-well plate, and transfection was performed when the cells reached 50% growth. The cells were transfected for 24, 48, and 72 h afterward. Then, 10 µL CCK-8 reagent was added to each well, and the plate was incubated for 0.5–4 h at 37 °C in a humidified atmosphere of 5% CO_2_ in accordance with the manufacturer’s instructions. The optical density for each well was measured using a microplate reader (Thermo, Pudong, Shanghai, China) at 450 nm.

### 4.6. EDU Proliferation Assay

The EDU cell proliferation assay was performed following the instructions provided in the Cell-Light™ EDU Apollo In Vitro Kit (Ribobio, Guangzhou, Guangdong, China). Briefly, the prepared EDU solution was added to the cell culture medium and incubated for 2 h. After removing the culture medium, the cells were fixed with cell fixation solution for 30 min, followed by permeabilization with permeabilization buffer for 10 min and washing with PBS. Subsequently, the cells were stained with Apollo dye and washed with PBS. Finally, the cell nuclei were stained with Hoechst dye.

### 4.7. Cell Apoptosis Analysis

Cell apoptosis was analyzed via flow cytometry (BD, Franklin, NJ, USA) using an Annexin V-fluorescein isothiocyanate (FITC)/propidium iodide (PI) Apoptosis Detection Kit (Vazyme, Nanjing, Jiangsu, China). Briefly, cells were transfected for 48 h, digested with 0.25% trypsin-ethylenediaminetetraacetic acid (Gibco, Grand island, NY, USA), collected through centrifugation, washed twice with cold Dulbecco’s phosphate-buffered saline (DPBS) (Gibco, Shunyi district, Beijing, China), and centrifuged at 4 °C. Then, DPBS was discarded. Next, 100 µL 1× Binding Buffer was added to each sample and mixed well. Then, 5 µL of FITC and PI were added separately, the mixture was incubated for 15 min at room temperature, and then 400 µL of 1× Binding Buffer was added. After the addition of 1× Binding Buffer, the mixture was mixed well. The samples were assayed via flow cytometry within 1 h. All data were analyzed using FlowJo software (version V10.8.1).

### 4.8. Western Blot

Protein expression levels in cells were detected using Western blot. Total protein was extracted from KGN cells using radioimmunoprecipitation assay lysis buffer I (Sangon Biotech, Songjiang, Shanghai, China) at 72 h after transfection, and its concentration was quantified using the Modified BCA Protein Assay kit (Sangon Biotech, Songjiang, Shanghai, China). The concentration of protein samples was kept constant through the addition of PBS to the protein sample. Then, the 4× protein sodium dodecyl sulfate–polyacrylamide gel electrophoresis (SDS-PAGE) loading buffer (Takara, Beijing, China) was added to each protein sample, and the samples were denatured for 5 min at 100 °C. Denatured proteins were resolved through 10% SDS-PAGE and electrophoretically transferred to a 0.45 µm polyvinylidene difluoride (PVDF) membrane (Sigma, Tullagreen, Carrigtwohill, Ireland). After 5 min of blocking with QuickBlock™ Blocking Buffer for Western blot (Beyotime, Shanghai, China), the PVDF membrane was incubated with the first antibody overnight at 4 °C, followed by rinsing three times for 5 min each rinsing. Next, the membrane was incubated with the corresponding secondary antibody for 1 h at room temperature. After rinsing with Tris-buffered saline and Tween 20 three times for 5 min each time, the chemiluminescence horseradish peroxidase substrate (Invitrogen, Carlsbad, CA, USA) was used to visualize the intensity of labeled proteins.

### 4.9. Dual-Luciferase Reporter Assay

The wild-type and mutant 3′UTR sequences of GHSR were cloned into the pmirGLO vector containing the luciferase reporter gene. The constructed luciferase plasmid (the pmirGLO plasmid containing miR-128-3p at the binding site of the GHSR 3′UTR or a mutation at the binding site) was cotransfected with miR-128-3p mimics into 293T cells. After 48 h, luciferase activity was detected using a dual-luciferase reporter assay kit (Vazyme, Nanjing, China) following the manufacturer’s instructions.

### 4.10. Statistical Analysis

All experiments were performed at least thrice. Comparisons between two and among multiple groups were performed using *t*-test and one-way variance analysis, respectively. All data were expressed as the mean ± SD. GraphPad Prism (version 9.0) was used for all analyses (GraphPad Software, La Jolla, CA, USA). *p* values < 0.05 were considered statistically significant.

## 5. Conclusions

**Figure 7:**
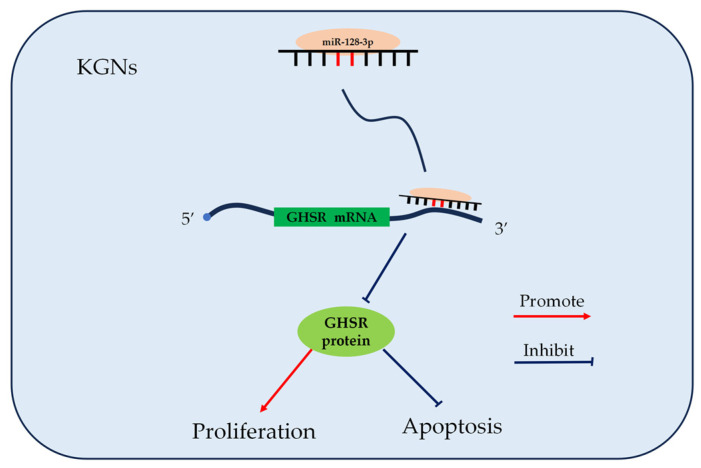
Schematic of miR-128-3p regulating the proliferation and apoptosis of KGN.

In vitro, miR-128-3p can target GHSR to regulate the expression of PCNA, BAX, and BCL2 genes and thereby impede KGN cell proliferation and promote KGN cell apoptosis (Figure 7).

## Data Availability

The data presented in this study are available upon request from the corresponding authors.

## Supplementary Materials

The supporting information can be downloaded at https://www.mdpi.com/article/10.3390/ijms25052720/s1.
